# Data element mapping to analyze fit for use of three XML standards for health workforce tracking

**DOI:** 10.1186/s12960-021-00615-x

**Published:** 2021-06-05

**Authors:** Amy Opalek

**Affiliations:** ECFMG|FAIMER, 3624 Market Street, PA 19104 Philadelphia, United States of America

**Keywords:** International data standard, Health workforce planning, Professional regulation, Interoperability, Health HRIS

## Abstract

**Background:**

Ensuring a sufficient supply and distribution of health care professionals is essential to meeting public health needs. Regulatory agencies protect their communities by ensuring that new health professionals have the required qualifications to practice safely and by tracking the volume and distribution of those professionals on an ongoing basis. The speed and accuracy of sharing these data could be greatly improved through the adoption of a data standard for information about health professionals. To date, however, no internationally accepted standard has emerged for this purpose.

**Purpose:**

This study examines three existing XML standards designed for the representation of individual worker data to determine if, and to what degree, each could be used for the tracking of health professionals.

**Methods:**

The data elements of the Europass schema, the HR Open Standard Recruiting specification, and the MedBiquitous Healthcare Professional Profile standard were fully examined and matching elements were mapped to the 200+ elements identified from a prior content analysis as required by a sample of 20 international regulatory agencies.

**Results:**

None of the schemas examined addressed more than half of the information elements required by regulators. All three schemas are found lacking in some key areas of interest, especially vital information that could disqualify ineligible applicant practitioners.

**Conclusions:**

The three standards could all be improved by including new elements essential to regulatory agencies. Regulatory agencies should be consulted in the development of new standards for representing potentially disqualifying information about candidates for professional practice.

## Background

Health professional regulatory agencies (HPRAs) serve key functions that contribute to the health of their communities. First, they serve as gatekeepers to health practice by verifying each health professional has the character and educational qualifications to practice safely. Second, they track professionals’ ongoing employment status and location to ensure a supply and distribution of health workers that is sufficient to meet their communities’ healthcare needs. Both of these tasks are essential to maintaining qualified health workforce of appropriate size, a key public health indicator [1]. Unfortunately, while many HPRAs may be adequately equipped to serve as effective gatekeepers for entry to practice, many are unable to accurately count or track their health human resources on an ongoing basis [2], due to insufficient, non-standardized, or nonexistent health human resource information systems.

The increasingly globalized nature of health and health care has accelerated the complications of effective health professions regulation in a variety of ways. It has been observed that the international mobility of skilled workers in general has been increasing [3]. Federations of nations in Europe and Southeast Asia have established policies for the mutual recognition of qualifications [4, 5], which could accelerate professional mobility within some geographical regions. Many countries continue to be heavily dependent upon foreign-trained health workers, with these workers comprising up to half of the local workforce [6, 7]. Projections indicating increasing demands for health workers globally [8] suggest that this trend will continue or worsen in the future. This dependency on foreign-trained workers complicates both the gatekeeping and monitoring roles of HPRAs. Regulators in source countries are faced with the difficulty of monitoring and accounting for the loss of their skilled professionals to other nations [9], and regulators in those destination countries face challenges assessing the adequacy of a professional’s educational qualifications when those qualifications were obtained in an unfamiliar educational system and documented in a foreign language. Sadly, in some countries where the licensure/registration process for a foreign-trained health worker can take months or even years [10], that worker’s vital skills may go unutilized. Finally, despite increasing use, telehealth continues to pose legal and ethical issues for regulators, especially when telehealth systems are not standardized [11, 12].

These trends toward a smaller, more tightly connected world put ever-increasing pressures on localized regulatory agencies in need of accurate, timely data about their health workforce. These pressures are exacerbated by a lack of standardized information systems to accurately track health workers and their qualifications. Processes for matching individuals across federated systems have been recommended [13], but research to date has mostly focused on patients, and does not include much of the information needed by HPRAs monitoring practitioners. While use of and research on health human resource information systems has been expanding recently [14, 15], a review of the literature revealed no studies comparing systems across countries [16]. Systems developed locally without reference to an endorsed standard run the risk of producing data that lack interoperability, making international data sharing and workforce analysis impractical or impossible, and contributing to further delays in the verification of health professionals’ qualifications.

The purpose of this research was to determine whether any existing data standards for describing professionals and their qualifications are sufficient to meet the needs of those who track health professionals internationally. Agencies responsible for the regulation of health professionals obtain data regarding the number and distribution of health workers in their jurisdiction via professionals’ initial applications and through ongoing, periodic re-licensure activities. The application forms used by medical regulatory agencies (MRAs) are openly accessible data collection tools that clearly demonstrate the type, scope, and format of information collected by agencies responsible for ensuring physicians’ competence. This research compares data elements extracted from an international sample of these documents with three existing standards which could be used for health workforce tracking. All three standards were published using Extensible Markup Language (XML), a commonly used language for expressing data schemas in a hierarchical structure.

## Methods

A purposive sample of the licensure/registration application forms for 20 international MRAs were collected to examine what information elements they require regarding health professionals. Each required data element was itemized and mapped in a matrix under categories including demographic information, educational history, employment history, certifications, licenses, personal references, and details of any adverse actions. The detailed results of this content analysis are reported in a prior study [17]. For this investigation, the full list of 200+ compiled data elements were mapped to the elements of three different XML schemas/standards used for representing individuals and their professional qualifications. The three standards examined were the Europass schema [18], the HR Open Standard Recruiting specification [19], and the MedBiquitous Healthcare Professional Profile standard [20]. Matching elements were classified as either exact or close matches. (An example of a close match is age and date of birth.) The data element matching was reviewed for accuracy by two individuals with expertise in informatics and health professions education. Each standard’s coverage of elements required by MRAs was compared, and elements that were poorly represented across all standards were identified.

## Results

A prior document analysis of physician licensure/registration application forms yielded over 200 distinct data elements required by MRAs [17]. These data elements represented information in the following facets of interest: demographics, contact information, education history (including pre-professional, professional, and continuing professional education), professional certification, examination results, licensure/registration history, employment history, personal references, criminal/civil claim history, disciplinary actions (and other unusual events), applicant health history, and professional insurance. Matches for each element were sought in each of the three existing standards used for describing professionals and their qualifications. The following sections describe the findings within each facet of information.

### Health professional demographics and contact information

Demographic and contact information are essential for accurately identifying and communicating with health professionals. These information elements may also be used to assess whether the demographic makeup of the health workforce matches that of the community, and to predict exits from the workforce based on typical retirement age. All three of the XML schemas reviewed contained elements for the professional’s name, date of birth, gender, nationality, and native language. None of the schemas contained elements for capturing the following details found on MRA application forms: date the applicant obtained legal permanent resident status, expiration date of applicant’s identification (passport, etc.), information about the applicant’s spouse beyond their name (spouse’s nationality, occupation, employer, registration status, professional ID number), or applicant’s designated next of kin. Further, none of the schemas provided a way for applicants to indicate their status as retired from practice. The HR Open schema had the greatest number of demographic elements available, including several that were not found in the MRA application forms, such as eye color, identifying marks, and blood type. The Europass schema had the fewest number of elements in this facet.

### Educational history

Information describing applicants’ educational history, whether pre-professional, professional, or continuing professional development, is essential to determining their qualifications. The data elements required by MRAs related to educational history were the most numerous of all facets. These could be grouped into three sub-facets: elements describing the educational institution, elements describing the program undertaken by the applicant at that institution, and elements describing the applicant’s experience in that program. Information elements describing the educational institution that did not appear in any of the three candidate schemas were: prior names of the institution, name and location of the parent institution (i.e., a medical college’s parent university), and the age of the institution. Program-level details that were not found in any of the schemas were: requirements for entry into the program, the language of instruction, and the standard duration of the program (as opposed to the length of time taken by this individual to complete the program). All schemas contained elements for capturing the applicant’s dates of attendance and degree title. Only the Europass schema contained an element which could be used to identify specific topics or skills addressed within the curriculum of a program. The HR Open schema was the only schema that contained elements for capturing the institution’s phone number or email address, or the name and contact information of specific individuals at the institution (e.g., the dean or registrar). Only the MedBiquitous schema contained elements for representing the accreditation status of the institution.

### Certification history

Many national systems rely on documentation of postgraduate education as the primary form of evidence of having achieved competence in a medical specialty. In some countries, such as the United States, professional certification is the more common way of demonstrating competence as a medical specialist. Consequently, it was not surprising that the two schemas developed in the US contained facets dedicated to the representation of professional certifications. By contrast, the Europass schema represented certifications as one of several types of *Achievement* for which supporting documents could be attached. Unlike the HR Open and MedBiquitous schemas, the Europass schema did not include elements for capturing information about the issuing agency or the date the qualification was awarded.

### Examination history

Several of the MRA application forms analyzed contained questions pertaining to the applicant’s performance on one of two types of exams: professional exams used for licensure or certification and assessments of language proficiency. The Europass schema models language proficiency as part of a Skills facet, which contains elements for sharing the name of the awarding body and date of award. The HR Open schema contains a PersonCompetency facet with a CompetencyEvidence subfacet that contains a CompetencyEvidenceType which may have a value of *assessment*. However, neither schema’s structure allows for the sharing of information describing certain characteristics of the assessments required by some MRAs, such as the exam type (formative or summative) or modality (oral, written, etc.).

### Employment history

The majority of MRAs require applicants to provide information about their current employment, past employment, and/or prospective employment. All three of the schemas reviewed contained facets for describing a professional’s current and prior employment, including the employer name and address, job description, and dates of employment. The MedBiquitous schema has an additional detailed structure for capturing information about any academic appointments held by the professional. None of the schemas captured the primary language(s) used by the professional during instances of employment.

### Licensure/registration history

The majority of MRA application forms require applicants to report any current or prior licenses or registration held in other jurisdictions. Only the HR Open and MedBiquitous schemas included facets for describing a professional’s registration or licensure, including details describing the issuing agency and the type and duration of the qualification. Only the MedBiquitous schema contained explicit elements for defining the jurisdiction and profession to which the license/registration permits access. One element of interest to MRAs that was not present in any schema was whether an exam was required for the license/registration.

### Professional references

Several MRAs required applicants to provide the name and contact information of individuals who could attest to the skills and character of the applicant. Only the HR Open schema provided elements for capturing data from or about personal references. Defined elements include the referee’s name, contact information, relationship to the applicant, number of years known, and comments. However, several elements required by MRAs were not addressed. These included the referee’s qualifications, native language, dates during which the applicant and referee worked together, and any potential conflicts of interest.

### Adverse/unusual events

On many MRA application forms, several lengthy items are dedicated to inquiring about unusual events in the applicant’s educational, employment, and licensure history that could be cause for concern. These events include official reprimands, fines, restrictions placed on practice, suspension or revocation of licensure or certification, termination of employment, and leaves of absence. The HR Open and MedBiquitous schemas each contained elements for describing some of these types of events (*violations* in the HR Open schema and *DisciplinaryInfo* in MedBiquitous). However, these schemas only presented these elements as options within the context of licensure/registration. Actions taken by educational institutions, employers, certifying agencies, and other organizations were not supported in any schema. Some of the more ambiguous types of events of interest to MRAs, such of leaves of absence, unsupported/unproven accusations of misconduct, and expunged disciplinary history, were also not supported.

### Criminal/civil claim history

In addition to the adverse actions described above, many MRAs inquire about an applicant’s criminal and civil claim history, including formal accusations of malpractice. Unfortunately, none of the three schemas examined provided elements for sharing information about a professional’s criminal or civil claim history.

### Applicant health

A third facet of potentially disqualifying information sought by MRAs relates to the applicant’s physical and mental health. Only the HR Open schema included data elements for describing a professional’s health in the form of a *disability* entity. Related elements include the type of disability, effective dates, and comments. Elements requested by MRAs that were not addressed by the schema were the name and contact information of the practitioner from whom the applicant receives treatment for this disability, explicit permission to contact that practitioner, and an indication of whether or not the disability impacts the professional’s ability to practice. Another health-related issue not addressed by any of the schemas was the professional’s inoculation history.

### Holistic comparison

In addition to the data elements described above, all schemas provided mechanisms for the attachment of supporting documentation across facets. However, there were other areas where the use of common objects across facets could be improved. In all schemas, improvements could be made in the internal consistency of use of common objects—*types* in XML parlance—such as institutions and people. The HR Open made the best use of a common *organization* type, which could be used to capture information about educational institutions, certifying agencies, employers, etc. MedBiquitous also had an *InstitutionInfo* type that was used in several facets, but not in all places where institutional information was captured (e.g., *DisciplinaryEntity*). All three schemas utilized types for representing addresses and other contact information consistently across multiple facets.

**Fig. 1:**
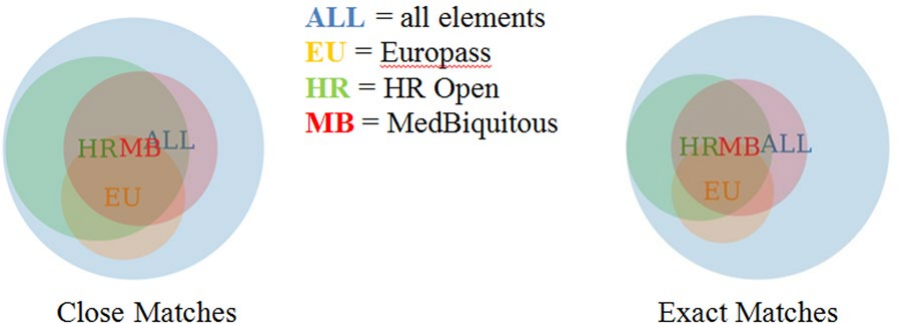
Data element coverage of three standards, close and exact matches

Looking at all of the data elements together, no schema/standard captured more than half of the elements identified as required by MRAs. Venn diagrams illustrating the relative mappings of elements in each standard with those required by MRAs, both close and exact matches, are presented in Fig. 1 below.

Overall, the HR Open schema supported the greatest number of elements in terms of exact (49.5%) and close (30.9%) matches, followed by the MedBiquitous Professional Profile schema (exact: 34.8%; close: 27.0%) and the Europass schema (exact, 25.5%; close, 15.2%). Elements not supported by any schema included those related to continuing professional development activities, performance on professional exams (certification or licensure), criminal history, adverse actions taken by organizations other than licensing authorities (e.g., schools or certifying agencies), professional insurance held, and details of the applicant’s health and inoculation history.

Each schema contained some elements not covered by the other two schemas. For example, the Europass schema contained a detailed representation of language ability not captured by the other standards. The HR Open schema contained many additional demographic elements not found in other schemas and was the only schema to accommodate personal references. The MedBiquitous schema provided structures for describing an institution’s accreditation.

## Discussion

The development of international data standards is essential to the exchange, comparability, and analysis of global health data and to the timely licensure and recognition of migrant health workers. This examination of three existing XML standards for the sharing of professional profile data finds that while many core data elements of several important information facets are addressed, each schema is lacking when it comes to information required by those who regulate health professionals and monitor their workforce. The standards are most notably lacking in the areas of potentially disqualifying information, including the professional’s criminal history, adverse actions taken by regulatory and other agencies, and the applicant’s health history. While the majority of practitioners will not have data to share in these areas, it is extremely vital that HPRAs receive accurate and timely information about those who do.

In addition to identifying information coverage of three XML schemas used for describing professionals and their qualifications, a useful byproduct of this analysis has been the creation of a crosswalk matrix among large portions of these schemas (see Appendix A). Agencies that have already built systems in line with one of these standards can use such a crosswalk to translate their data into other standards’ schemas and to merge data from sources encoded in different schemas. The developers of these and other standards could also use this analysis to inform future updates to their own schemas.

## Conclusions

In an era of increasing professional mobility and cross-border healthcare, it is imperative to adopt internationally acceptable standards for the representation of health professionals and their qualifications. This has become even more apparent recently, as many parts of the world have been affected by a refugee crisis, a global health emergency, and increasing usage of telemedicine. The current pandemic has highlighted the need for international collaborations among health regulators and health workers supported by a shared understanding of and reliance on professional skills and competencies. These recent global crises have also put telemedicine in the spotlight, showcasing the advantages of being able to deliver health care at a distance. International borders have become more permeable, or perhaps less relevant, to the provision of health care. The walls separating our data, however, remain opaque.

The standardization of representation of health professionals internationally also has implications for the evaluation of health care quality. The systematic, accurate identification of treating health professionals across EHR systems may allow for a more nuanced analysis of the factors contributing to patient health. Connecting patient outcomes to individual practitioner or practitioner team characteristics, including the educational experiences of those practitioners, could provide valuable guidance to educators of the next generation of health professionals.

As pressures from confederations of nations encourage standards in professional education and the development of more advanced regulatory frameworks in some countries, this is a prime opportunity to incorporate international data standards. This research can be used to inform the revision of existing standards or the creation of new standards for constructs currently not well supported, such as adverse actions and activities associated with continuing professional development. If international MRAs and partner agencies, such as universities and certifying agencies, are to adopt a standard for sharing data about health professionals, there is still much work to be done.

## Data Availability

The data sets analyzed in this study (three published XML standards) are available from their respective publishers as noted in the references. The data generated from this analysis are included in the manuscript.

## Appendix A: Data element mappings by facet

The tables below show data elements mapped among the three schemas examined. Only those elements that appear in more than one schema are presented. Italicized text indicates the nature of a close (non-exact) match.

### Health professional demographics and contact information

**Table:**

Data element	Europass	HR open	MedBiquitous
Current name	Identification\PersonName	Person\name	Name\FormattedName
Prior/alternate name		PersonName\alias	Name\FormerName; Name\Alias
Mother's Name		PersonLegal\motherName *Surname only*	PersonalInfo\FamilyMember where relationship = mother
Father's name		PersonLegal\fatherName *Surname only*	PersonalInfo\FamilyMember where relationship = father
ID number		Person\legalId\value; PersonLegal\passportID	PersonalInfo\NationalID
Date of birth	Identification\Demographics\Birthdate	Person\birthDate	PersonalInfo\BirthDate
Place of birth		Person\birthPlace	PersonalInfo\CountryOfBirth
Gender	Identification\Demographics\Gender	Person\gender	PersonalInfo\Gender
Race/Ethnicity		PersonLegal\race; Person\ethnicity	PersonalInfo\Race; PersonalInfo\Ethnicity
Nationality	Identification\Demographics\NationalityList	PersonLegal\nationality; Person\citizenship	PersonalInfo\Citizenship
Country of residence		Person\residenceCountry	PersonalInfo\CountryOfResidence
Languages spoken	Skills\Linguistic\MotherTongueList; Skills\Linguistic\ForeignLanguageList	Person\primaryLanguage *Secondary languages not represented*	PersonalInfo\Language
Address	Identification\ContactInfo\Address	Person\communication\address	Address
Telephone number	Identification\ContactInfo\TelephoneList	Person\communication\phone	PersonalInfo\ContactNumber\CountryCode + PersonalInfo\ContactNumber\TelephoneNumber + PersonalInfo\ContactNumber\Extension where Description = Phone
Email address	Identification\ContactInfo\Email	Person\communication\email	PersonalInfo\EmailAddress

### Education history

**Table:**

Data element	Europass	HR open (EducationAttendance\+)	MedBiquitous (EducationInfo\+)
Institution name	Education\Organization\Name	Institution\name	InstitutionInfo\InstitutionName
Institution ID number		Institution\id	InstitutionInfo\InstitutionID
Institution address	Education\Organization\ContactInfo\Address	Institution\communication\address	InstitutionInfo\Address
Program/discipline name	Education\Description *Broader term*	Programs	ProgramName
Education level	Education\Level	Institution\educationLevelCodes	
Education start date	Education\Period\From	Start	StartDate
Education end date	Education\Period\To	End	EndDate
Degree/diploma title	Education\Title	EducationDegrees\name	Degree\label
Abbreviation of degree title	Education\Title		Degree
Degree/diploma issue date		EducationDegrees\date	CompletionDocumentIssuedDate
Degree classification, distinction, or honors	Achievement\Title Where label = honors_awards	EducationDegrees\academicHonors	Distinction

### Employment history

**Table:**

Data element	Europass (WorkExperience\+)	HR open (EmployerHistory\+)	MedBiquitous (OccupationInfo\+)
Employer name	Employer\Name	Organization\name	InstitutionInfo\InstitutionName
Employer address	Employer\ContactInfo\Address	Organization\communication\address	InstitutionInfo\InstitutionAddress
Employer telephone number	Employer\ContactInfo\TelephoneList	Organization\communication\phone	
Employer Email	Employer\ContactInfo\Email	Organization\communication\email	
Employment sector	Employer\Sector	Organization\ownershipTypeCode	
Job title		PositionHistories\title	OccupationTitle
Specialty	Position [OccupationalFieldType]		Specialty
Job description	Description	PositionHistories\descriptions	OccupationInfo\Occupation
Employment status [past, current, prospective]	Period\Current *“prospective” not supported*	Current *“Prospective” not supported*	OccupationStatus *“prospective” not supported*
Employment start date	Period\From	Start	StartDate
Employment end date	Period\To	End	EndDate
Employment duration	Period\To–Period\From *sum of* WorkExperience *instances may or may not match*	End–start *sum of* position *instances may or may not match*	StartDate–EndDate *sum of* Occupation *instances may or may not match*

### Examinations/assessments

**Table:**

Data element	Europass (*for language assessments only*) Skills\+	HR open
Exam name	Linguistic\ForeignLanguageList\VerifiedBy\Certificate\Title	PersonCompetency\competencyEvidence\evidenceName
Exam agency	Linguistic\ForeignLanguageList\VerifiedBy\Certificate\AwardingBody	PersonCompetency\competencyEvidence\verificationSourceName
Exam subject	Linguistic\ForeignLanguageList\VerifiedBy\Certificate\Title	PersonCompetency\competencyName
Exam level	Linguistic\ForeignLanguageList\VerifiedBy\Certificate\Level	PersonCompetency\competencyEvidence\descriptions
Exam date	Linguistic\ForeignLanguageList\VerifiedBy\Certificate\Date	PersonCompetency\competencyEvidence\effectivePeriod

### History of licensure and registration

**Table:**

Data element	HR open (License\+)	MedBiquitous (License\+)
MRA name	IssuingAuthority	LicensureEntity\LicensureEntityName
MRA address	IssuingAuthority\communication\address	LicensureEntity\ContactInformation\Address
MRA phone number	IssuingAuthority\communication\phone	LicensureEntity\ContactInformation\ContactNumber
MRA email address	IssuingAuthority\communication\email	LicensureEntity\ContactInformation\EmailAddress
Profession	Name *Name of profession may be included in license name*	Profession
License/Reg type	Type	LicenseCategory
License/Reg status	Status	LicenseStatus
License/Reg start date	FirstIssued	InitialLicensureDate
License/Reg end date	EffectiveTimePeriod *End date could be derived from this value* + *firstIssued*	LicenseExpirationDate

### Certifications

**Table:**

Required data element	Europass	HR open (Certification\+)	MedBiquitous (CertificationInfo\+)
Certifying agency name		issuingAuthority	CertificationBoard
Medical specialty		Descriptions *broader term*	CertificateInfo\CertificateName
Certification title	Achievement\Title where label = certifications	Name	CertificateInfo\CertificateName
Certification date		firstIssued	CertificateIssuance\CertificateIssueDate

### Adverse actions related to licensure/registration

**Table:**

Data element	HR open (License\+)	MedBiquitous (DisciplinaryInfo\+)
Event type	Violations\item\value; Restrictions\item\value	BoardAction\ActionDescription
Event status	Violations\item\validFrom + Violations\item\validTo; Vestrictions\item\validFrom + Restrictions\item\validTo *Not all statuses can be inferred from dates*	BoardAction\ActionSpan BoardAction\ActionStayed BoardAction\IndefiniteAction
Acting agency	IssuingAuthority	DisciplinaryEntity
Acting agency location	IssuingAuthority\communication\address	DisciplinaryEntity\ContactInformation
Event date	Violations\item\validFrom; Restrictions\item\validFrom	DisciplinaryInfo\OrderDate; DisciplinaryInfo\OrderEffectiveDate
Action summary	Violations\item\description; Restrictions\item\description	BoardAction

## Abbreviations

EHR: Electronic health record
HPRA: Health professional regulatory authority
HRIS: Human resource information system
MRA: Medical regulatory authority
XML: Extensible Markup Language
